# Genetic influences on insight problem solving: the role of catechol-*O*-methyltransferase (*COMT*) gene polymorphisms

**DOI:** 10.3389/fpsyg.2015.01569

**Published:** 2015-10-13

**Authors:** Weili Jiang, Siyuan Shang, Yanjie Su

**Affiliations:** Department of Psychology and Beijing Key Laboratory of Behavior and Mental Health, Peking UniversityBeijing, China

**Keywords:** creativity, insight problem solving, *COMT* gene, catecholamines, gender differences

## Abstract

People may experience an “aha” moment, when suddenly realizing a solution of a puzzling problem. This experience is called insight problem solving. Several findings suggest that catecholamine-related genes may contribute to insight problem solving, among which the catechol-*O*-methyltransferase (*COMT*) gene is the most promising candidate. The current study examined 753 healthy individuals to determine the associations between 7 candidate single nucleotide polymorphisms on the *COMT* gene and insight problem-solving performance, while considering gender differences. The results showed that individuals carrying A allele of rs4680 or T allele of rs4633 scored significantly higher on insight problem-solving tasks, and the *COMT* gene rs5993883 combined with gender interacted with correct solutions of insight problems, specifically showing that this gene only influenced insight problem-solving performance in males. This study presents the first investigation of the genetic impact on insight problem solving and provides evidence that highlights the role that the *COMT* gene plays in insight problem solving.

## Introduction

In daily life, people may get stuck with some problems that cannot be solved using regular methods, and suddenly somehow get the solution or an idea, accompanying with a delightful “aha” feeling. This experience is called insight problem solving (Sternberg and Davidson, 1995; Smith and Kounios, 1996; Bowden et al., 2005; Chu and MacGregor, 2011). Insight problem solving, in contrast with other forms of problem solving, is an “all-at-once” process rather than an analytic process or a trial-and-error process, solvers usually cannot report how they get insight solutions. Insight problem solving is critical to individual survival, well-being and prosperity, for it may occur in several domains, from perception to language comprehension, like recognizing an ambiguous object or understanding a joke (Kounios and Beeman, 2014). Moreover, insight problem solving can result in important innovations that contribute to human society development and achievement (Simonton, 2003; Runco, 2004). For example, Archimedes’ principle was discovered because of a light flashing in Archimedes’ mind when he got into a tub and saw the water overflowing, crying “Eureka”. Despite its importance, the underlying biological mechanisms of insight problem solving are not yet completely understood. Insight problem solving is not a unique human capacity – it has also been found in non-human species (Shettleworth, 2012), such as chimpanzees (Köhler, 1925) and elephants (Foerder et al., 2011). This suggests that this type of problem solving has deep evolutionary and biological roots. Further, insight problem solving is often regarded as a form of creativity (Friedman and Förster, 2005), and twin studies demonstrate that creativity has genetic underpinnings (Velázquez et al., 2015). Most molecular genetic studies of creativity adopted divergent thinking tasks rather than insight problem tasks (e.g., Reuter et al., 2006; Runco et al., 2011), while these two tasks were used to test two different components of creativity (Lin and Lien, 2013). Taken together, it is essential to investigate the deep genetic influences on insight problem solving, to improve our understanding of insight problem solving and creativity.

Previous cognitive and neuroscience studies of insight problem solving indicate that catecholamines, including dopamine (DA) and norepinephrine (NE), may play a critical role in the cognitive process of insight problem solving (Folley et al., 2003; Heilman et al., 2003; Cai et al., 2009). Insight problem solving benefits from positive mood states, especially those activating positive moods rather than deactivating ones, like happiness (Isen et al., 1987; Baas et al., 2008; Subramaniam et al., 2009). Further, insight problem solving process involves some aspects of executive function, such as working memory (Fleck, 2008; Gilhooly and Fioratou, 2009; Lin and Lien, 2013) and cognitive flexibility (Lin et al., 2014). Meanwhile, both positive moods and executive functions, in turn, are related to the catecholamine levels (Ashby et al., 1999; Chudasama and Robbins, 2006; Hillier et al., 2006; Baas et al., 2008; Subramaniam et al., 2009; Cools and D’Esposito, 2011). Furthermore, findings in psychiatry demonstrate that some kinds of mental states that are associated with DA level can lead to positive consequence in terms of insight problem solving. For instance, schizophrenia, a disease that results from hyperactive DA signal transduction (Davis et al., 1991), is correlated with better insight problem-solving performance (Karimi et al., 2007).

Hence, genes involved in catecholamine transmission are likely to be the candidate genes that may contribute to insight problem solving. Of the catecholamine-related genes, catechol-*O*-methyltransferase (*COMT*) gene is the most investigated gene, which encodes the COMT enzyme. The COMT enzyme is the most important catabolic enzymes, especially in the prefrontal cortex, which degrades the catecholamines (Chen et al., 2004). Through encoding COMT enzyme, *COMT* genes influence the level of DA and NE.

The *COMT* gene is located on chromosome 22q11 in humans. The greatest variance of COMT activity is explained by a common single nucleotide polymorphism (SNP) Val158Met (rs4680). The G→A transition makes amino acid valine (Val) be replaced by methionine (Met), and the activity of COMT enzyme will decrease by approximately 35–50% (Lachman et al., 1996; Chen et al., 2004). As a result, G/G (Val/Val) gene carriers have the highest activity of COMT enzyme and the lowest level of DA and NE. Previous studies used divergent thinking tasks to investigate the genetic influences on creativity, and found that the A allele of rs4680 is associated with better creativity (Runco et al., 2011; Zhang et al., 2014a). For example, using the Instance Task and Realistic Creative Problem Solving, researchers found that individuals with A/A genotype showed better divergent thinking (Runco et al., 2011). And insight problem solving has high correlation with divergent thinking (Gilhooly and Murphy, 2005; DeYoung et al., 2008; Lee and Therriault, 2013). On the other hand, studies have found that carriers of the A/A genotypes in rs4680 have the best performance on executive functions, like working memory and cognitive flexibility (Egan et al., 2001; Goldberg et al., 2003; Witte and Flöel, 2012), which play an important role in insight problem solving (Fleck, 2008; Gilhooly and Fioratou, 2009; Lin and Lien, 2013; Lin et al., 2014). For example, Barnett et al. (2007b) conducted a meta-analysis of a specific executive function task – the Wisconsin Card Sorting Test and found that individuals with the A/A genotype performed better in the Wisconsin Card Sorting Test than those with the G/G genotype. Furthermore, from the psychiatric perspective, in Chinese population, individuals with genotype G/G show the lowest Schizotypal Personality Questionnaire scores (Ma et al., 2007), which have been shown to be related to decreased insight problem-solving performance (Karimi et al., 2007). Besides the *COMT* gene rs4680, there are other *COMT* gene functional SNPs that might have an impact on insight problem solving. For instance, SNP rs5993883 is associated with divergent thinking (Zhang et al., 2014a).

In addition, gender may moderate the relation between the *COMT* gene and insight problem solving. COMT effects are not the same between different genders (Harrison and Tunbridge, 2008). For men, COMT activity in prefrontal cortex is 17% higher than that in women (Chen et al., 2004), and the *COMT* gene has a greater impact in men. For example, the A allele of rs4680 was associated with better working memory only in men, not in women (Barnett et al., 2007a).

In all, insight problem solving is a very important creative ability found not only in humans but also in non-human animals. The *COMT* gene had been assessed with divergent thinking tasks, but not with this kind of creative task. In addition, studies mainly focus on a common SNP of the *COMT* gene, rs4680. So the present study aimed to investigate a possible association between insight problem solving and the *COMT* gene, including several SNPs rather than only rs4680 to look at the broader picture of *COMT* gene influences, and considering gender as a potential moderator.

## Materials and Methods

### Participants

The sample comprised 753 healthy Chinese high-school students (516 females and 237 males) in Southwest China. The mean age was 16.54 years, *SD* = 0.70 years, range from 14.33 to 19.14 years. The study was approved by the local ethics committees of Peking University. All participants gave written informed consent after a description and an explanation of the study. Upon completion of all tests, reimbursements were given for their participation. Participants first completed a paper-and-pencil measurement, and then their buccal cells were collected for genotyping.

### Assessment of Insight Problem Solving

Participants were required to solve 13 classic, pure insight problems, which were adapted from prior published researches (Dow and Mayer, 2004; Chiu et al., 2008; DeYoung et al., 2008). Pure insight problems can only be solved by insight solution rather than trial-and-error solution. Problems without confusion or eliminate were chosen, which covered a range of difficulty. Example 1: Fang and Hong were born on the same day of the same month of the same year to the same mother and the same father – yet they are not twins. How is that possible? The correct answer is that they are triplets or multiple births. Example 2: There exists an ancient invention still used in the world today, which allows people to see through the wall. What is it? The correct answer is windows. For each problem, participants were given 2 min to solve. After being tested, participants were asked whether they knew the problem and the solution before. Responses were scored for accuracy on unknown problems.

### Genotyping

A saliva swab was obtained from each subject and DNA was extracted using FlexiGene DNA Kit (Qiagen, Valencia, CA, USA). Based on previous studies, seven single polymorphisms in *COMT* gene (rs2020917, rs737865, rs59938883, rs4633, rs6267, rs4818, and rs4680) were chosen in the Hapmap database^1^ and NCBI SNP database^2^. All these SNPs are reported having an effect on catecholamines, with minor allele frequency (MAF) > 5%. The SNP genotyping work was performed using an improved multiplex ligation detection reaction (iMLDR) technique developed by Genesky Biotechnologies Inc. (Shanghai, China). A multiplex PCR-ligase detection reaction method was used in the iMLDR. For each SNP, the alleles were distinguished by different fluorescent labels of allele-specific oligonucleotide probe pairs. Different SNPs were further distinguished by different extended lengths at the 3′end. All primers, probes and labeling oligos were designed by and ordered from Genesky Biotechnologies Inc. (Shanghai, China). The raw data was analyzed by GeneMapper 4.1. For quality control, a random DNA sample accounting for 5% was genotyped twice, yielding a reproducibility of 100%.

### Statistical Analysis

First, descriptive statistics were ascertained to examine the distributional properties of the average number of correct solution of insight problems, and how this performance correlated with age. To determine whether the average number of correctly solved insight problems differed by gender, *t*-tests were also employed. Second, Hardy–Weinberg equilibrium, linkage disequilibrium (LD) and haplotype blocks were tested. Third, descriptive statistics for insight problem-solving performance by genotype in females and males were conducted. Finally, the genotype of seven SNPs of the *COMT* gene and gender interaction with insight problem solving were analyzed using ANCOVA analysis, taking age as a covariate variable. We reported uncorrected *P*-values in the text.

## Results

The average number of correctly answered insight problems was 7.28 (*SD* = 2.54, *range* = 1–13). This outcome significantly correlated with age (*r* = 0.12, *p* = 0.004), and so we controlled for the effect of age in the following analysis. Additionally, we found a gender difference, with males (*M* = 7.72, *SD* = 2.50) showing higher insight problem-solving scores compared to females (*M* = 7.08, *SD* = 2.54), *t*(751) = 3.24, *p* = 0.001, *d* = 0.25.

**FIGURE 1:**
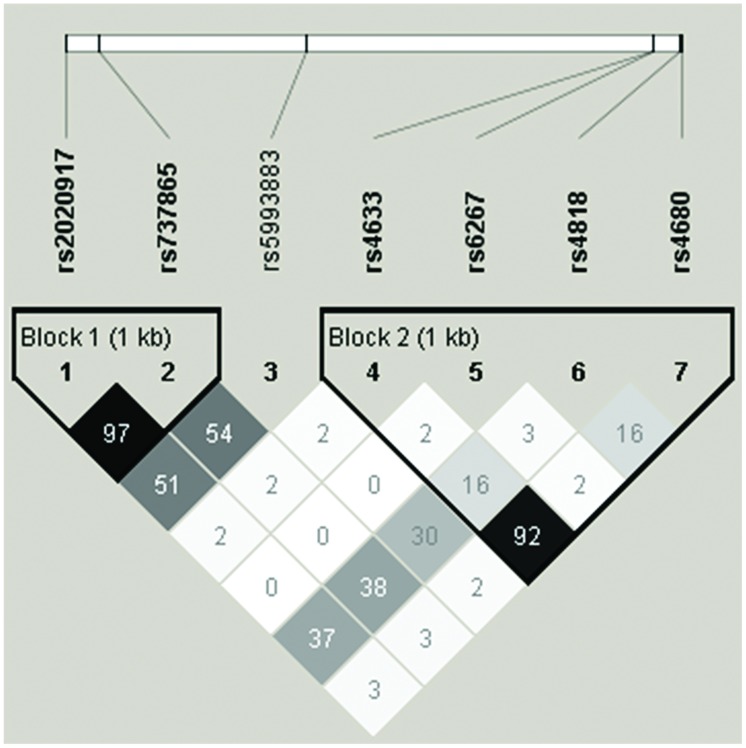
**Linkage disequilibrium (LD) pattern of the seven catechol-*O*-methyltransferase *(COMT)* single nucleotide polymorphisms (SNPs) analyzed in the present study**. The upper panel shows the location of seven polymorphisms in *COMT* gene and the lower panel presents the output of Haploview. Numbers in each square present the *r*^2^ value of a pairwise LD relationship between any two SNPs. Two haplotype blocks were defined using the solid spine of LD algorithm.

**Table 1** summarizes the minor allele frequencies, the number of participants per allelic group, and *p*-values for the Hardy–Weinberg equilibrium tests. All seven SNPs were polymorphic with MAF > 5% and in Hardy–Weinberg equilibrium. Haploview was used to analyze LD and construct haplotype blocks of the studied *COMT* polymorphisms. **Figure 1** shows LD pattern of the seven *COMT* SNPs. Two haplotype blocks were detected from these seven SNPs using the solid spine of LD algorithm. Block 1 was composed of rs2020917 and rs737865, while block 2 included four SNPs (rs4633, rs6267, rs4818, rs4680).

**Table 1:** Gene location, allele, and genotype frequencies of the investigated *COMT* gene polymorphisms.

Single nucleotide polymorphisms (SNP)^a^	Position^b^	Location	Allele (minor/major)	Genotype	*n*	Frequency	MAF^c^ (%)	*p*-HWE^d^
rs2020917	18308884	5′upstream	T/C	CC/CT/TT	386/310/57	0.513/0.412/0.076	0.282	0.629
rs737865	18310121	Intron1	C/T	TT/TC/CC	390/309/54	0.518/0.410/0.072	0.277	0.497
rs5993883	18317638	Intron 1	G/T	TT/TG/GG	278/348/107	0.369/0.462/0.142	0.383	0.910
rs4633	18330235	Exon 3	T/C	CC/CT/TT	420/290/43	0.558/0.385/0.057	0.250	0.443
rs6267	18330263	Exon 3	T/G	GG/GT/TT	666/85/2	0.884/0.113/0.003	0.059	0.680
rs4818	18331207	Exon 4	G/C	CC/CG/GG	340/328/85	0.452/0.436/0.113	0.331	0.661
rs4680	18331271	Exon 4	A/G	GG/GA/AA	424/281/48	0.563/0.373/0.064	0.250	0.875

*^a^SNPs are listed down the column in sequential order from the 5′ end to the 3′ end of the sense strand of COMT*.

*^b^Physical position is based on NCBI B36*.

*^c^MAF, minor allele frequency*.

*^d^*p*-HWE, *p*-value of Hardy–Weinberg equilibrium test*.

There were no significant differences in the distribution of the seven SNPs between males and females. **Table 2** shows the descriptive statistics for insight problem-solving performance by gender and different SNPs. As the number of participants in the least common allele pair was less than 20, for rs2020917, rs737865, rs4633, rs6267, and rs4680, we collapsed the less common homozygote carriers and heterozygote carriers into one group for further ANCOVA analysis. The interactions between the seven SNPs in the *COMT* gene and gender on insight problem-solving performance are shown in **Table 3**. We found that three *COMT* gene SNPs (rs5993883, rs4633, rs4680) displayed an impact on insight problem solving, whilst the other four SNPs (rs2020917, rs737865, rs6267, rs4818) in the *COMT* gene had no significant effect on insight problem solving.

**Table 2:** Insight problem-solving performance by genotype and gender.

		Total		Males		Females	
		*n*	*M (SD)*	*n*	*M (SD)*	*n*	*M (SD)*
rs2020917	C/C	386	7.31 (2.50)	123	7.89 (2.36)	263	7.04 (2.51)
	C/T	310	7.21 (2.57)	99	7.50 (2.64)	211	7.08 (2.54)
	T/T	57	7.46 (2.73)	15	7.80 (2.73)	42	7.33 (2.75)
rs737865	A/A	390	7.30 (2.49)	126	7.88 (2.34)	264	7.03 (2.51)
	A/G	309	7.21 (2.58)	96	7.50 (2.68)	213	7.08 (2.54)
	G/G	54	7.56 (2.74)	15	7.80 (2.73)	39	7.46 (2.77)
rs5993883	G/G	107	7.22 (2.69)	29	6.90 (2.68)	78	7.33 (2.70)
	T/G	348	7.23 (2.55)	108	7.50 (2.48)	240	7.11 (2.58)
	T/T	278	7.32 (2.49)	92	8.08 (2.41)	186	6.95 (2.45)
rs4633	C/C	420	7.11 (2.55)	132	7.46 (2.51)	288	6.95 (2.55)
	C/T	290	7.52 (2.51)	89	7.99 (2.54)	201	7.31 (2.48)
	T/T	43	7.33 (2.67)	16	8.38 (2.03)	27	6.70 (2.84)
rs6267	G/G	666	7.32 (2.51)	211	7.78 (2.50)	455	7.11 (2.50)
	G/T	85	6.94 (2.77)	25	7.20 (2.55)	60	6.83 (2.88)
	T/T	2	7.50 (0.71)	1	8.00 (–)	1	7.00 (–)
rs4818	C/C	340	7.45 (2.36)	108	8.09 (2.33)	232	7.15 (2.46)
	C/G	328	7.19 (2.63)	102	7.50 (2.62)	226	7.04 (2.63)
	G/G	85	6.98 (2.51)	27	7.07 (2.56)	58	6.93 (2.51)
rs4680	A/A	48	7.56 (2.61)	19	8.63 (2.03)	29	6.86 (2.74)
	A/G	281	7.52 (2.49)	87	7.94 (2.46)	194	7.32 (2.49)
	G/G	424	7.09 (2.56)	131	7.44(2.56)	293	6.94 (2.55)

**Table 3:** ANCOVA results for the interaction of genotype and gender on insight problem-solving performance.

	Genotype × Gender		Genotype		Gender	
	*F*	*p* (*η^2^*)	*F*	*p* (*η^2^*)	*F*	*p* (*η^2^*)
rs2020917	1.18	0.279 (0.002)	0.56	0.454 (0.001)	**7.49**	**0.006 (0.010)**
rs737865	1.23	0.267 (0.002)	0.39	0.539 (0.001)	**7.35**	**0.007 (0.010)**
rs5993883	**3.83**	**0.022 (0.011)**	1.20	0.303 (0.003)	0.62	0.433 (0.001)
rs4633	0.48	0.489 (0.001)	**4.11**	**0.043 (0.006)**	**8.21**	**0.004 (0.011)**
rs6267	0.01	0.914 (<0.001)	1.28	0.258 (0.002)	3.26	0.071 (0.005)
rs4818	1.74	0.177 (0.005)	2.93	0.054 (0.008)	1.80	0.180 (0.003)
rs4680	0.45	0.503 (0.001)	**5.11**	**0.024 (0.007)**	**8.04**	**0.005 (0.011)**

*Bolded values means significant effect*.

Specifically, there was a significant main effect of *COMT* rs4633 on insight problem-solving tasks, *F*(1,708) = 4.11, *p* = 0.043, *η^2^* = 0.006; T allele (TT and CT) carriers had higher insight problem-solving scores than those with CC genotype (*d* = 0.15). Similarly, there was also a significant effect of *COMT* rs4680 on insight problem-solving performance, *F*(1,708) = 5.11, *p* = 0.024, *η^2^* = 0.007; A allele (AA and GA) carriers scored higher in insight tasks than those with GG genotype (*d* = 0.18).

**FIGURE 2:**
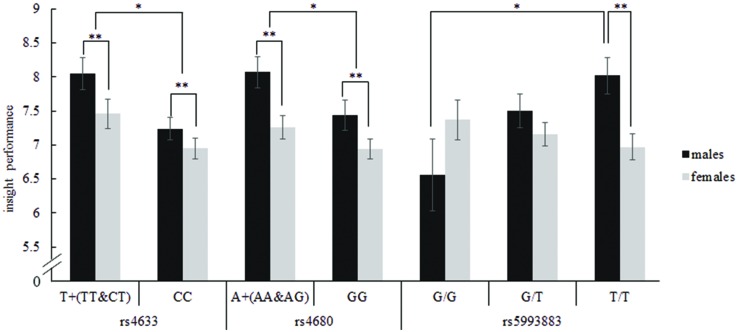
**Insight problem-solving performance depending on the interaction of *COMT* rs4633, rs4680, rs5993883, and gender**. For rs4633, T allele carriers had a higher score on insight tasks than CC carriers, and males performed better on insight problems than females. For rs4680, A allele carriers had a higher score on insight tasks than GG carriers, and males performed better on insight tasks than females. For rs5993883, in males, TT genotype carriers performed better on insight problem solving than GG carriers. Additionally, male carriers of TT genotype scored higher on insight tasks than females with TT. Error bars represent standard error. ^∗^*p* < 0.05, ^∗∗^ p < 0.01.

There was a significant interaction between *COMT* rs5993883 and gender on insight problem-solving tasks, *F*(2,687) = 3.83, *p* = 0.022, *η^2^* = 0.011 (see **Figure 2**). Simple effects tests showed that only in males did these three genotype carriers differ in insight problem-solving performance [*Bonferroni test, F*(2,687) = 3.19, *p* = 0.042, *η^2^* = 0.009]. Specifically, TT genotype carriers showed a better insight problem-solving performance than GG carriers only for males (*p* = 0.043, *d* = 0.58). Further, due to this interaction the general advantage of males vs. females was restricted to TT genotype carriers where males with TT genotype scored higher than females with TT [*Bonferroni test, F*(2,687) = 9.97, *p* = 0.002, *η^2^* = 0.014, *d* = 0.41]. Because of the large differences in sample sizes, with only 29 males carrying G/G genotypes, far less than other gene-gender-combination groups, to confirm the effect, we randomly chose 29 samples from the other five groups to conduct another ANCOVA analysis. The results didn’t change, for there still exists a significant interaction between genotype of rs5993883 and gender, *F*(2,168) = 4.63, *p* = 0.011, *η^2^* = 0.052. Although we found three *COMT* gene SNPs (rs5993883, rs4633, rs4680) displayed an impact on insight problem solving, none of the reported significance survived correction for multiple comparisons using false discovery rate method.

## Discussion

The present study tested the association between insight problem-solving performance and the *COMT* gene including seven SNPs, and found that *COMT* rs4680 and *COMT* rs4633 were correlated with insight problem solving. Moreover, there was a gender difference in the association between the *COMT* gene and insight problem solving, with only male carriers of the TT genotype in *COMT* rs5993883 showing better insight tasks performance than GG carriers, an effect not found in females. These findings supported the relationship between insight problem solving and the *COMT* gene, although the results should be interpreted cautiously. After all, at significant levels corrected for multiple comparisons, none of *COMT* gene variants influenced insight problem solving. However, based on previous studies, there were some indirect supporting evidences for the association between the *COMT* gene and insight problem solving. Combined with these evidence, the result of these multiple comparisons here might indicate that the effect size of this association is not large enough, although we couldn’t rule out the possibility that the association between the *COMT* gene and insight problem solving are not to be found.

*COMT* rs4680, the most commonly studied *COMT* gene polymorphisms, was found to be correlated with insight problem solving, which suggested that A allele carriers had better insight problem-solving abilities. This result is in line with some previous indirect findings that the A allele of *COMT* rs4680 plays an important role in executive functions like working memory, schizotypy and divergent thinking (Egan et al., 2001; Ma et al., 2007; Runco et al., 2011; Zhang et al., 2014a), which are all correlated with insight problem solving (Karimi et al., 2007; DeYoung et al., 2008; Fleck, 2008). Additionally, rs4633, which has a strong LD with rs4680, also showed an effect on insight problem solving, with T allele carriers achieving higher insight problem-solving scores. This result was also consistent with previous findings that rs4633 showed a significant association with schizophrenia (Handoko et al., 2005), which has been demonstrated to correlate with insight problem solving (Karimi et al., 2007). For rs5993883, gender moderated its relation with insight problem solving, and rs5993883 had an impact on insight problem solving only in males. In line with previous studies, the gender difference of the *COMT* effect is found in many domains, especially this male-specific effect, such as cognition (Harrison and Tunbridge, 2008), prefrontal blood oxygenation-level-dependent activation (Coman et al., 2010), and addiction-like behavior in rats (Tammimäki and Männistö, 2011). The mechanisms underlying the association between insight problem solving and the *COMT* gene may be related to the DA pathways and the noradrenergic system. COMT metabolizes all catecholamines, including DA and NE (Weinshilboum et al., 1999). Evidence shows that DA may affect insight problem solving through its effects on individuals’ working memory and cognitive flexibility (Gilhooly and Fioratou, 2009; Lin et al., 2014). Meanwhile, NE may significantly impact on cognitive flexibility, and therefore improve solving insight problems (Beversdorf et al., 1999; Hillier et al., 2006; Alexander et al., 2007). Future studies may further investigate levels of DA and NE along with some cognitive abilities at the same time to verify these two possible explanations.

Insight problem solving, measured by close-ended creative tasks, reflects a kind of convergent thinking. According to Guilford (1950), creativity involves two components: divergent thinking and convergent thinking. Divergent thinking refers to open-ended problem solving, making individuals generate multiple ideas or solutions. Previous studies have found that rs737865, rs4680, and rs5993883 of *COMT* genes were correlated with divergent thinking as well (Zhang et al., 2014a). In the current study, rs4633, rs4680, and rs5993883 of *COMT* genes were correlated with insight problem solving. These same genetic influences may indicate that divergent thinking and convergent thinking are related. Previous studies have also supported this from other perspectives. For example, both divergent thinking and insight problem solving benefited from positive mood states (Baas et al., 2008). Moreover, using transcranial direct current stimulation, studies have found that both divergent thinking and insight problem solving have a right hemispheric superiority (Chi and Snyder, 2011; Mayseless and Shamay-Tsoory, 2015). Some researchers even believed that divergent thinking was the foundation of insight problem solving (Brophy, 1998). However, other studies found that divergent thinking and convergent thinking involves different process (Chermahini and Hommel, 2010; Lin and Lien, 2013). Future studies may further investigate the relationship between divergent thinking and convergent thinking, especially from biological perspectives. In addition, insight problem solving is only one kind of convergent thinking task, and there are other tasks reflecting convergent thinking as well, for instance, the Remote Associates Task (RAT; Mednick, 1962). In fact, previous studies have found that DA is correlated with RAT scores (Chermahini and Hommel, 2010; Ueda et al., 2015). Future studies may investigate the relation between the *COMT* gene and RAT performance to draw a whole genetic impact picture of convergent thinking, and to further understand two components of creativity – divergent thinking and convergent thinking – from genetic perspectives.

There are limitations of this study. The current study only examined one catecholamine-related gene, the *COMT* gene, as a genetic influences on insight problem solving. However, there are many other important genes involved in catecholamine transmission that could contribute to insight problem solving, such as DA transporter (*DAT1*), DA D2 receptor gene (*DRD2*), DA D4 receptor gene (*DRD4*), and monoamine oxidase A gene (*MAOA*) (Bachner-Melman et al., 2005; Reuter et al., 2006; Runco et al., 2011; Mayseless et al., 2013; Zhang et al., 2014a,b). Moreover, divergent thinking has been found to have an association with other catecholamine-related genes. For example, carriers with 7R allele of DA receptor D4 gene (*DRD4*) performed worse in divergent thinking (Mayseless et al., 2013). Future studies may consider other single polymorphisms effects as well as gene–gene interaction effects on insight problem solving, to further understand the genetic impact on insight problem solving and creativity. Moreover, although participants were not asked whether they experienced an “aha” moment of insight, we adopted pure insight problem tasks that can only be solved through an insight process (Dow and Mayer, 2004; DeYoung et al., 2008). Future studies may add this inquiry to confirm the association between the *COMT* gene and insight.

## Conclusion

The present study provides the first evidence for the *COMT* gene as the genetic influences on insight problem solving, although the results should be interpreted with great caution. Despite the underlying mechanisms are still unclear, the current study demonstrated that the *COMT* gene does indeed contributes to insight problem solving, and its effects are modulated by gender. This research may promote our understanding of the evolutionary and biological roots of insight problem solving.

## Author Contributions

WJ, YS contributed to the conception and design of the work. WJ, SS collected and analyzed the data. WJ, SS, YS contributed to the writing of the manuscript.

## Conflict of Interest Statement

The authors declare that the research was conducted in the absence of any commercial or financial relationships that could be construed as a potential conflict of interest.

## Footnotes

1 http://hapmap.ncbi.nlm.nih.gov/

2 http://www.ncbi.nlm.nih.gov/SNP.
